# Counting touching wheat grains in images based on elliptical approximation

**DOI:** 10.18699/vjgb-25-64

**Published:** 2025-07

**Authors:** D.R. Avzalov, E.G. Komyshev, D.A. Afonnikov

**Affiliations:** Institute of Cytology and Genetics of the Siberian Branch of the Russian Academy of Sciences, Novosibirsk, Russia; Institute of Cytology and Genetics of the Siberian Branch of the Russian Academy of Sciences, Novosibirsk, Russia Kurchatov Genomic Center of ICG SB RAS, Novosibirsk, Russia; Institute of Cytology and Genetics of the Siberian Branch of the Russian Academy of Sciences, Novosibirsk, Russia Novosibirsk State University, Novosibirsk, Russia Kurchatov Genomic Center of ICG SB RAS, Novosibirsk, Russia

**Keywords:** wheat, grains, counting, digital images, segmentation, algorithm, concave points, пшеница, зерна, подсчет, цифровые изображения, сегментация, алгоритм, угловые точки

## Abstract

The number of grains of a cereal plant characterizes its yield, while grain size and shape are closely related to its weight. To estimate the number of grains, their shape and size, digital image analysis is now generally used. The grains in such images may be completely separated, touching or densely packed. In the first case, the simplest binarization/segmentation algorithms, such as the watershed algorithm, can achieve high accuracy in segmentation and counting grains in an image. However, in the case of touching grains, simple machine vision algorithms may lead to inaccuracies in determining the contours of individual grains. Therefore, methods for accurately determining the contours of individual grains when they are in contact are relevant. One approach is based on the search for pixels of the grain contact area, in particular, by identification of concave points on the grain contour boundary. However, some grains may have chips, depressions and bulges, which leads to the identification of the corner points that do not correspond to the grain contact region. Additional data processing is required to avoid these errors. In this paper, we propose an algorithm for the identification of wheat grains in an image and determine their boundaries in the case when they are touching. The algorithm is based on using a modification of the concave point search algorithm and utilizes a method of assigning contour boundary pixels to a single grain based on approximation of grain contours by ellipses. We have shown that the proposed algorithm can identify grains in the image more accurately compared to the algorithm without such approximation and the watershed algorithm. However, the time cost for such an algorithm is significant and grows rapidly with increasing number of grains and contours including multiple grains.

## Abstract (ru)

Количество зерен растения напрямую характеризует его урожайность, а размер и форма тесно связаны с массой семян. Для оценки количества зерен, их формы и размеров в настоящее время, как правило, используют анализ цифровых изображений. Зерна на таких изображениях могут быть полностью разделены, соприкасаться или быть плотно упакованными. В случае разделенных зерен высокую точность выделения и подсчета зерен на изображении позволяют получить самые простые алгоритмы бинаризации/сегментации, например алгоритм водораздела. Но в случае соприкасающихся зерен простые алгоритмы машинного зрения могут приводить к неточностям в определении контуров отдельных зерен. В этой связи актуальными являются методы точного определения контуров индивидуальных зерен в случае их соприкосновения. Один из подходов основан на поиске пикселей области соприкосновения зерен, в частности с помощью поиска угловых точек на границе контура зерен. Однако зерна могут иметь сколы, впадины и выпуклости, что приводит к идентификации угловых точек, которые не соответствуют области контакта зерен. Это влечет за собой ошибки и для их устранения требует дополнительной обработки данных, фильтрации ложных угловых точек. В настоящей работе мы предлагаем алгоритм идентификации зерен пшеницы на изображении, который позволяет идентифицировать касающиеся зерна и определять их границы на изображении. Он базируется на модификации алгоритма поиска угловых точек и использует метод отнесения пикселей границы контура к одному зерну на основе аппроксимации контуров зерен эллипсами. Мы показали на тестовых изображениях, что предложенный алгоритм позволяет более точно идентифицировать зерна на изображении по сравнению с алгоритмом без такой аппроксимации и алгоритмом водораздела. Однако временные затраты для такого алгоритма существенны и быстро растут с увеличением количества зерен и контуров, включающих несколько зерен.

## Introduction

One of the most important areas of cereal breeding and genetic research is identification of genes that control yield. The number of grains of a cereal plant directly characterizes its yield, and the grain size and shape are closely related to its weight (Zhang X. et al., 2014; Brinton, Uauy, 2019). Estimation of the number of grains, their shape and size is frequently performed using high-performance phenotyping (Afonnikov et al., 2016; Li et al., 2020; Kolhar, Jagtap, 2023), which is based on digital image analysis (Tanabata et al., 2012; Whan et al., 2014; Komyshev et al., 2017). These methods are characterized by high performance, low equipment costs and simplicity of image acquisition protocols. An additional advantage of these methods in comparison with manual counting is that it is possible to accurately determine not only the number of grains, but also their characteristics (size, shape, color) (Tanabata et al., 2012; Cervantes et al., 2016; Komyshev et al., 2020), which is difficult or impossible in the case of manual counting. Furthermore, the obtained images can be stored without changes, while grain characteristics may vary depending on the duration of storage (Afonikov et al., 2022).

Typical images for analysis are grains on a light background obtained using a digital camera, desktop scanner, or smartphone (Herridge et al., 2011; Tanabata et al., 2012; Whan et al., 2014; Komyshev et al., 2017). The grains in such images may be completely separated, touching, or densely packed. In case of separated grains, the simplest binarization/segmentation algorithms can be used to isolate grains in the image, for example, the watershed algorithm (Roerdink, Meijster, 2000). In this case, the size and shape of each grain can be estimated (Mebatsion et al., 2013). However, such a protocol requires a significant amount of time spent on careful placement of grains, which makes it difficult to analyze a large number of samples. In the case of dense packing, it is difficult to determine the contours of individual grains due to possible overlap, and an objective assessment can only be expected for the number of grains in the image, not for their size and shape. In the case of touching grains, simple machine vision algorithms can lead to inaccuracies in determining the contours of individual grains, but the shape and size characteristics of individual grains can be evaluated. This protocol does not require careful placement of grains on the surface, which reduces the time spent on analysis. In this regard, the development of methods for determining the contours of individual grains in the case of their contact in images is relevant.

To solve this problem, approaches based on machine vision (Wang, Paliwal, 2006; Qin et al., 2013) and deep learning (Yang et al., 2021) have been developed. Deep learning methods are currently being actively developed, but they require large samples of images marked manually for training, which is labor-intensive. Machine vision algorithms are less demanding on the size of training samples and their annotations, and are also being actively developed (Liang et al., 2022; Lin et al., 2023). To extract grains, they use binarization algorithms to detect grain contours and subsequent shape analysis for complex contours that include two or more grains. One of the approaches used for analyzing complex contours is based on finding pixels in the grain contact area. To search for such pixels, an algorithm is used to find corner points on the grain contour boundary (Gao et al., 2017; Liu et al., 2017; Tan et al., 2019; Liang et al., 2022; Zhang J. et al., 2022). This algorithm allows to quickly identify points where the contour boundary bends sharply. Pixels where the curvature of the contour line is greatest are considered potential points of grain contact. However, grains may have chips, cavities, and bumps, which leads to the identification of corner points that do not correspond to the grain contact area. This leads to errors, fixing which requires additional data processing to filter out false corner points.

In this paper, an algorithm is proposed for identification of wheat grains in the image, which allows us to identify touching grains and determine their boundaries in the image. It is based on a modification of the corner point search algorithm and uses the method of assigning pixels of the contour boundary to a single grain based on the approximation of grain contours by ellipses.

## Materials and methods

**Fig. 1.**
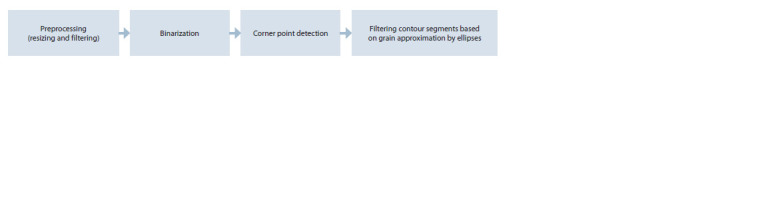
The main stages of image processing for counting wheat grains.

Identification of grain contours in an image. The general scheme of the image processing method is shown in Figure 1. It includes the following steps: • Image preprocessing (size reduction and Gaussian filter). • Binarization (separation of the areas of grains and the background). • Search for corner points in the grain contours. • Post-processing of contour pixels based on ellipse approximation.

Each of the stages involved the use of various computer vision algorithms described below.

The high resolution of the original images (3,968×2,976 pixels) negatively affected the running time of computer vision algorithms. During the preprocessing step, the image resolution was reduced to 1,984×1,488 pixels. It was found empirically that downsizing reduces the running time of the algorithms by several times without significant loss in the grain counting accuracy. For this purpose, a method based on bilinear interpolation was used (Gonzalez, Woods, 2004). In the original image, non-overlapping windows with a size of 2×2 pixels were replaced by one pixel in the converted image. The intensity values of the red, green, and blue components of this pixel were calculated based on the corresponding pixel components of the input window.

Let (xi, yj), i, j = 1, 2, be 2×2 neighboring pixels with coordinates (xi, yj), f (xi, yj) is the color component intensity for pixel i, j. The color intensity for the resulting pixel in the downsized image f (x, y) was calculated using the following algorithm: 1. Linear interpolation in the X direction:

**Formula. 1.**
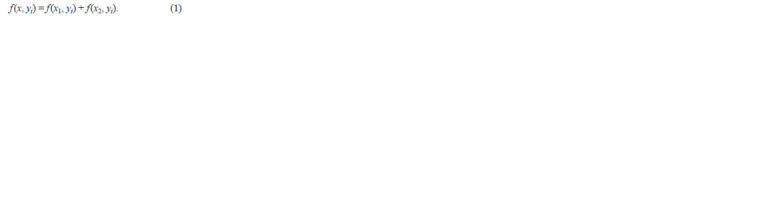
Formula. 1.

2. Linear interpolation of the obtained values in the Y direction:

**Formula. 2.**
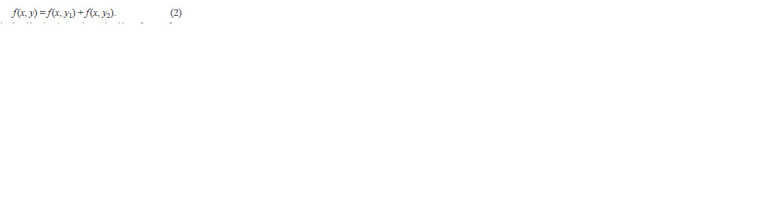
Formula. 2.

The image obtained by (1, 2) was downsized by a factor of 2.

A Gaussian filter (Gedraite, Hadad, 2011) was applied to the downsized image to eliminate noise. The filtered image was converted to HSV color space. This transformation improves the identification of the differences between the grains and the background (Fisenko V.T., Fisenko T.Yu., 2008; Domasev, Gnatyuk, 2009).

Smoothing by the mean shift algorithm (Comaniciu, Meer, 1999) was performed at the next step of image preprocessing. The image was converted to grayscale and binarized using the Otsu algorithm (Otsu, 1979).

**Fig. 2.**
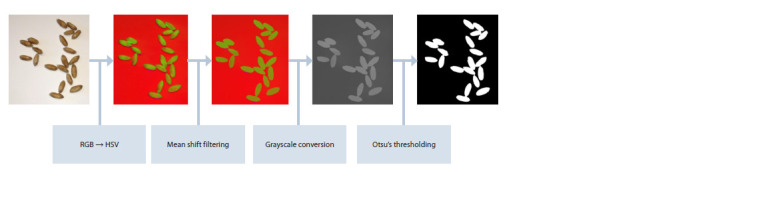
Results of preprocessing and binarization of grain images.

The results of preprocessing and binarization for a scaled image after Gaussian filtering are shown in Figure 2.

Contour analysis and corner pixel identification. Contours corresponding to the grain regions were identified in the binary masked image. A contour is a curve that connects all pixels of the edge of the grain area in a binarized image. Each contour was analyzed independently.

The contour boundary is traversed; for each pixel p of the boundary, the value of the corner response function, CRF, was calculated (Tan et al., 2019):

**Formula. 3.**
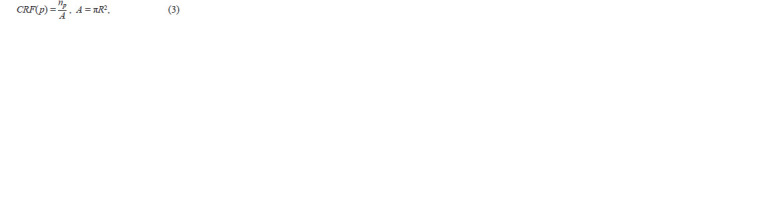
Formula. 3.

where np is the number of pixels that belong to a contour inside a circle with radius R and center at pixel p, and A is the total number of pixels in this circle (Fig. 3а). The value R = 7 was selected empirically from the set [3, 4, …, 10] yielding the best accuracy values (at R > 10, the algorithm performance decreased substantially). The value of this function is close to ~0.5 on the “straight” part of the grain contour boundary. The large fraction of grain pixels inside the circle (large CRF value) indicates pixels belonging to the corner (Fig. 3b, c).

**Fig. 3.**
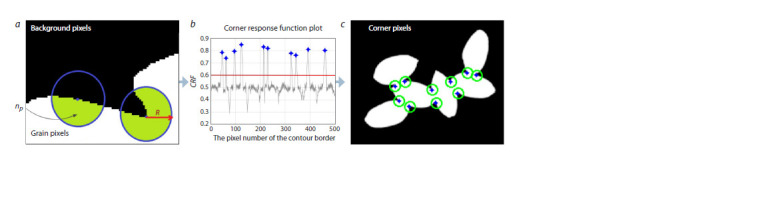
Using the corner response function to identify the corner pixels of the contour. a, Visualization of the CRF calculation: background pixels are displayed in black, grain pixels outside and inside the CRF circle are displayed in white and green, respectively; b, the CRF plot (Y-axis) for contour pixels (X-axis), the peaks corresponding to the corner points are shown by blue diamonds; c, corner pixels shown in the binarized image (blue diamonds inside green circles).

The pixel was determined as a corner point if CRF > 0.6 (Zhang J. et al., 2022). The number of grains within the contour could be estimated using the number of corner points Ncorners and the number of contour boundary segments between corner points Rclosed as Ngrains = Ncorners /2 – Rclosed + 1 (Liu et al., 2017). The disadvantage of the algorithm lies in the assumption of an elliptical grain contour. In real-world examples, false corner points can be detected due to chips and irregularities on the grain surface.

**Fig. 4.**
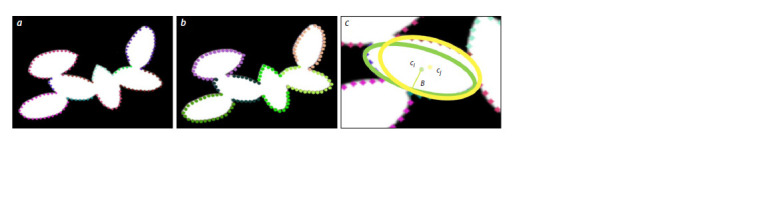
The elliptical approximation algorithm used to identify contour boundary segments belonging to the same grain. a, Pixels belonging to the same segment of the boundary between corners are shown in the same color; b, pixels of the contour belonging to the same grain are shown in the same color; c, alternate location of two ellipses for segments of the same grain, their centers ci and cj is shown as yellow and green dots, the axis of ellipse B is shown.

Approximation of grain contours by ellipses. Since grains have a shape close to elliptical, they can be approximated by ellipses, which helps breaking the contour of several grains into segments belonging to the same grain even if there are some false corners. The problem is to split the set of segments into subsets in such a way that each subset represents segments of the border of one grain (Fig. 4a, b).

The algorithm was based on finding the optimal partitioning of contour segments belonging to ellipses, minimizing the partitioning error. The partitioning error is the total error of pixel approximation of each of their ellipses. For some grains, several ellipses can be inscribed in its contour segments (Fig. 4c). Therefore, a penalty was added to the error value to choose from several inscribed ellipses the one that has the center position closest to the average center.

The contour was described by a set of pixels pik, i = 1, …, mk. The coordinates of ellipse pixels were represented by quadratic form a11x2 + 2a12 xy + a22 y2 + 2b1x + 2b2 y + 1 = 0.

The coordinates of each pixel were substituted in the equation representing the equation system:

**Formula. 4.**
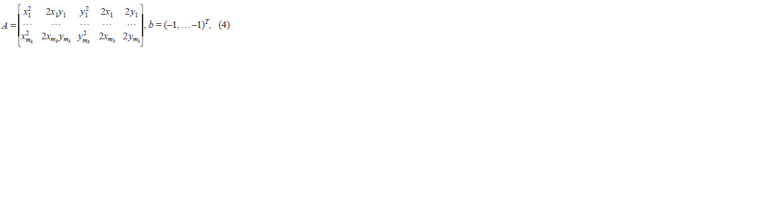
Formula. 4.

**Formula. 5.**
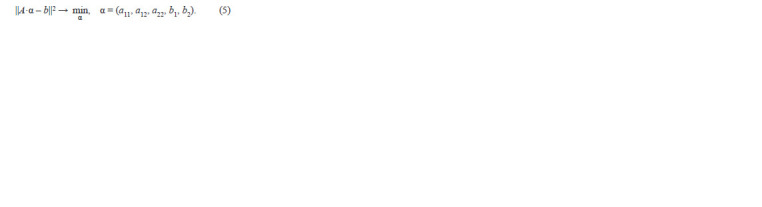
Formula. 5.

The optimal solution can be found using the least squares method and SVD decomposition (Brinton, Uauy, 2019). The solution to α* can be found as:

**Formula. 6.**
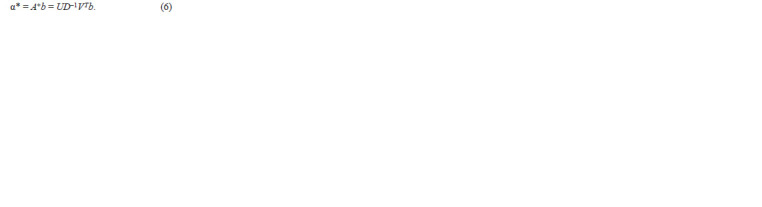
Formula. 6.

In this case, the total partitioning error was calculated using the following formula:

**Formula. 7.**
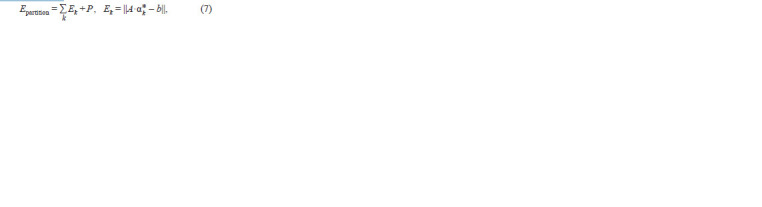
Formula. 7.

where

**Formula. 8.**
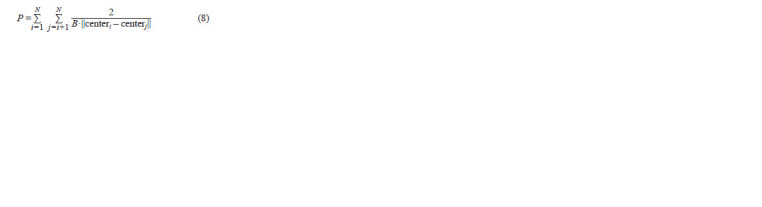
Formula. 8.

is the penalty for “incorrect” partitioning, and B is the minimum axis length of the inscribed ellipses (Fig. 4с).

**Fig. 5.**
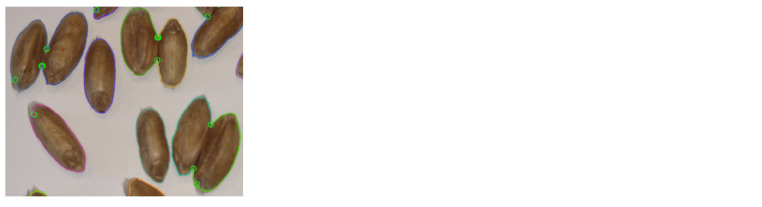
The result of splitting the contours of several grain groups based on the ellipse approximation algorithm The identified corner pixels are shown as green circles. Contour pixels that belong to the same grain are shown in the same color.

As a result of finding the partition of the contour with the lowest error, the number of grains is equal to the number of subsets in the partition. This approach solves the problem with extra corner points, which affects the accuracy of the method. An example of the result of such an algorithm when identifying grains is shown in Figure 5.

Figure 5 demonstrates the success of the algorithm application. Two touching grains in the lower right corner (the turquoise and green colors of the contours) were split correctly despite the extra corner pixel for the right grain.

The described algorithms were implemented in Python v.3.9, using the OpenCV v. 4.6.0 (Howse, 2013) and Numpy v. 1.21 libraries (https://numpy.org/).

**Evaluation of the accuracy of grain identification in an image.** For each image, the number of grains was found using the described algorithm. For each image, the accuracy of the algorithm was estimated using the following metric:

**Formula. 9.**
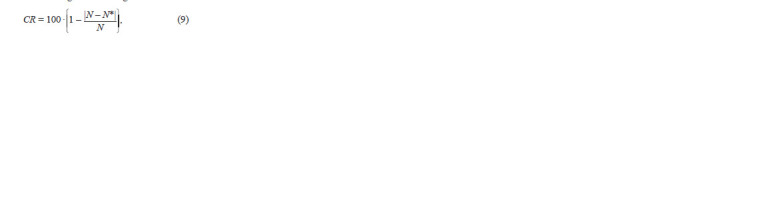
Formula. 9.

where N* is the number of grains determined using the proposed algorithm, and N is the true number of grains. Two accuracy measures were calculated for contour analysis: CRcp for the algorithm without correction by ellipses and CRcpe for the algorithm with correction by inscribed ellipses.

Additionally, we estimated the average accuracy for grain identification images for the erosion (CRe) and watershed CRw methods (Zhang J. et al., 2022). Additionally, the time for image processing, T, was estimated for the algorithm with correction using inscribed ellipses. Calculations were performed on a laptop with an Intel i5 4 * 2.9 GHz processor and 6 GB of RAM running the Windows 10 operating system.

## Results and discussion

**Fig. 6.**
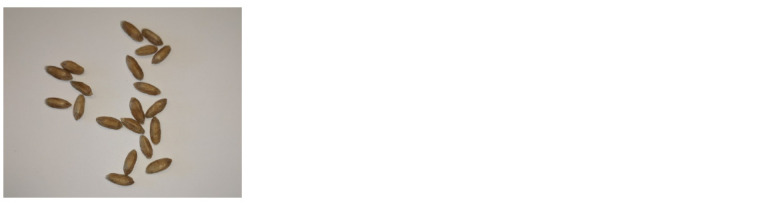
Example of an image of wheat grains in contact.

A set of 9 images of wheat grains on a white sheet of paper was used for analysis. Images were obtained using a HUAWEI P20 smartphone with a Sony IMX380 camera and flash. Imaging was performed in auto mode, the high dynamic range (HDR) option was turned off. Grains in the image were placed loosely, but they had a significant number of contacts. An example of a grain image is shown in Figure 6

Six images had 20 grains each; the sample also included images of 31, 46, and 51 grains.

The Table shows the results of counting the number of grains using two algorithms: using only corner points (cp) and using correction by the ellipse-based corner point algorithm (cpe). The Table shows estimates of the number of grains identified by the algorithm, as well as measures of accuracy and calculation time.

**Table 1.**
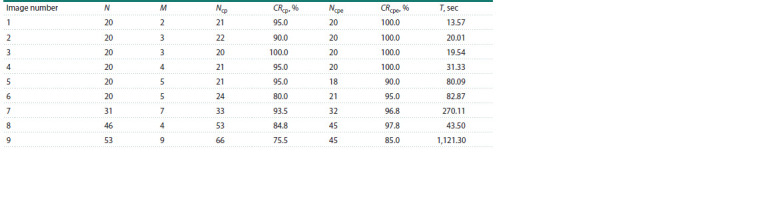
Accuracy of grain counting methods based on corner points without and with correction based on the ellipse method for 9 test images Notе. Columns represent the number of grains, N; the maximum number of grains in the contour, M; the number of grains determined by the cp algorithm, Ncp; the accuracy of the cp algorithm, CRcp; the number of grains determined by the cpe algorithm, Ncpe; the accuracy of the cpe algorithm CRcpe; the running time of the cpe algorithm, T (sec).

The average accuracy value for the cp algorithm was CRcp = 0.90, for the cpe algorithm CRcp = 0.96, for the watershed algorithm CRw = 0.77, and for the erosion algorithm CRe = 0.93. These data demonstrate that the ellipse-based grain number correction algorithm yields the most accurate results. However, the accuracy of the algorithm depends on the number of grains that form complex contours: the more grains in the contour, the lower the accuracy. The accuracy also depends on the number of grains, because for large numbers of grains, the probability of their contact is higher.

The correction of the seed detection algorithm by the ellipse-based method yielded performance comparable to some previously published methods based on corner points identification. Tan et al. (2019) used a corner point detection algorithm for counting rice grains in combination with a neural network with error back propagation for subsequent correction of segmentation results. On average, grain segmentation accuracy for different rice varieties was 94 %. Wang and Paliwal (2006) applied the watershed algorithm to seed images after segmentation and transformation depending on the distances between background and grain pixels. The algorithm was used to count grains in images for six types of plants (winter wheat, hard white-grain wheat, and hard amber wheat, barley, oats, and rye). The proportion of correctly identified grains ranged from 88.6 to 94.4 % for wheat and from 55.4 to 79.0 % for the other plants. Liu et al. (2017) suggested an algorithm based on detecting feature points in the image and estimating the correlation between their number and the number of grains in the image. The authors tested the algorithm for wheat and rice grains and showed that the error of their method was from 0 to 4.7 % (on average, 0.1 % for wheat and 1.5 % for rice), while applying the usual watershed algorithm led to an error of 14 to 40 % for wheat and of 20 to 50 % for rice seeds. Liang et al. (2022) proposed a comprehensive approach that identifies the number of non-touching grains by the K-means method, a layered watershed algorithm was used to identify and count rarely touching grains, and a dividing line algorithm was used for densely lying grains. The accuracy of the method was 99.65 %.

Thus, the corner point algorithm with ellipse-based correction in general yielded an accuracy comparable to existing algorithms (especially in the case when there are few contours with a large number of touching grains). However, the execution time of this algorithm increases quickly with increasing both the number of complex contours and the number of grains analyzed. For a number of grains more than 20 and a large number of contours with multiple grains, the time of execution becomes unacceptable for analysis. This occurs because with increasing number of contiguous grains in a single contour, the number of possible combinations of ellipse location subsets increases. Moreover, the number of contour pixels, the coordinates of which are used to compose the systems of linear equations (4), is growing. To reduce the execution time (in the case of the described implementation of the method), it is possible to parallelize the algorithm. Further optimization is possible by using estimates of the number of possible grains, for example, by the contour area. This will reduce the number of partitions.

Note also that the algorithm uses an approximation of the grain shape by ellipses, which may not be applicable to grains of a more complex shape, such as beans. However, in the general case, this algorithm allows to use an arbitrary shape of grain contours, which in the future can be implemented for grains of other plant species.

## Conclusion

An algorithm is proposed for identifying and counting grains in digital images in the case of their touching. The algorithm is based on binarization of the image to select contours containing grains and further processing of these contours. Processing consists of finding corner points on the contour and then selecting them by assigning pixels of the contour border to a single grain based on the approximation of grains by ellipses. The post-processing step allowed to exclude false regions of grain contacts in the contour. Analysis of the test images showed that in the case when the number of touching grains is small, the algorithm allows us to obtain a high accuracy of grain counting (up to 100 %, and systematically better than without ellipse-based approximation). However, when contours include a large number of touching grains, the execution time of the algorithm increases, which makes it impractical in analysis.

## Conflict of interest

The authors declare no conflict of interest.
